# Association between 24-hour blood pressure variability and chronic kidney disease: a cross-sectional analysis of African Americans participating in the Jackson heart study

**DOI:** 10.1186/s12882-015-0085-6

**Published:** 2015-06-18

**Authors:** Rikki M. Tanner, Daichi Shimbo, Albert W. Dreisbach, April P. Carson, Ervin R. Fox, Paul Muntner

**Affiliations:** Department of Epidemiology, University of Alabama at Birmingham, 1665 University Boulevard, Suite 230 J, Birmingham, AL 35294 USA; Columbia University Medical Center, New York, NY USA; Department of Medicine, University of Mississippi Medical Center, Jackson, MS USA

**Keywords:** Kidney disease, Blood pressure, Variability, ABPM, Health disparities

## Abstract

**Background:**

Studies suggest 24-h blood pressure (BP) variability has prognostic value for cardiovascular disease. Several factors associated with high 24-h BP variability are also common among individuals with chronic kidney disease (CKD). We hypothesized 24-h BP variability would be higher for individuals with versus without CKD.

**Methods:**

We analyzed 1,022 Jackson Heart Study participants who underwent ambulatory blood pressure monitoring (ABPM). Twenty-four hour BP variability was defined by two metrics: day-night standard deviation (SD_dn_) and average real variability (ARV). CKD was defined as ACR ≥30 mg/g or eGFR <60 mL/min/1.73 m^2^.

**Results:**

The mean SD_dn_ of systolic BP (SBP) was 10.2 ± 0.2 and 9.1 ± 0.1 mmHg and the mean ARV of SBP was 9.2 ± 0.2 and 8.6 ± 0.1 mmHg for those with and without CKD, respectively (each *p* ≤ 0.001). After adjustment for age and sex, SD_dn_ and ARV were 0.98 mmHg (95 % CI 0.59, 1.38) and 0.52 mmHg (95 % CI 0.18, 0.86), respectively, higher among participants with versus without CKD. These differences were not statistically significant after further multivariable adjustment including 24-h mean SBP. Older age, and higher total cholesterol and 24-h mean SBP were associated with higher SD_dn_ and ARV of SBP among participants with CKD. Mean SD_dn_ and ARV of diastolic BP (DBP) were higher for participants with versus without CKD but these associations were not present after multivariable adjustment.

**Conclusion:**

Data from the current study suggest that CKD is associated with higher 24-h BP variability, but the association is primarily explained by higher mean BP among those with CKD.

**Electronic supplementary material:**

The online version of this article (doi:10.1186/s12882-015-0085-6) contains supplementary material, which is available to authorized users.

## Background

Over 26 million American adults have chronic kidney disease (CKD) [1], evidenced by estimated glomerular filtration rate (eGFR) < 60 ml/min/1.73 m^2^ or albumin-to-creatinine ratio (ACR) ≥ 30 mg/g. CKD is a substantial public health challenge given its high prevalence and association with adverse outcomes, including cardiovascular disease (CVD) incidence and all-cause mortality [2, 3]. Identifying factors that explain this increased risk may provide guidance on the development of interventions to reduce it.

Ambulatory blood pressure monitoring (ABPM) is useful for the identification of phenotypes that cannot be ascertained by blood pressure (BP) measurements in the clinic setting. This includes the identification of white coat hypertension, masked hypertension, circadian BP patterns, and 24-h BP variability. Recent data have suggested that 24-h BP variability has prognostic value for CVD and mortality independent of mean BP [4–6]. For example, Hansen, et al. reported an association between higher 24-h BP variability and increased risk for cardiovascular mortality and stroke in a pooled analysis of 11 studies [4]. Autonomic dysfunction has been proposed as a key factor underlying higher 24-h BP variability [7]. Many individuals with CKD have autonomic dysfunction and other factors associated with high 24-h BP variability including older age, higher mean systolic BP (SBP), and higher levels of inflammation are common among individuals with CKD [8–10]. Therefore, we hypothesized that individuals with CKD would have higher 24-h BP variability compared to their counterparts without CKD. To test this hypothesis, we conducted an analysis of African-American adults participating in the Jackson Heart Study.

## Methods

### Study participants

The Jackson Heart Study is a community-based observational study designed to identify risk factors for CVD in African Americans. Details of the study design and recruitment have been published previously [11–13]. In brief, 5,301 African Americans ≥ 21 years of age were recruited from the Atherosclerosis Risk in Communities (ARIC) site in Jackson, Mississippi, and a representative sample of urban and rural Jackson tri-county (Hinds, Madison, and Rankin) residents, study volunteers, randomly contacted individuals, and secondary family members. Baseline data collection occurred between September 2000 and March 2004. The study protocol was approved by the Institutional Review Boards governing research in human subjects at the participating centers and all participants provided written consent.

### Data collection

Data used for the current analysis were collected through an in-home interview; a study examination after an overnight fast; and, for a subset of participants (*n* = 1,148), 24-h ABPM. Information on age, sex, education, income, cigarette smoking, and a history of diabetes, stroke, or myocardial infarction were collected during the study interview. During the clinic visit, a standardized protocol was followed to obtain two BP measurements, waist circumference was measured, and blood and urine samples were collected. Information was recorded on all medications, vitamins, mineral supplements, and herbal or home remedies used within the 2 weeks prior to the participant’s interview [12].

Using the blood and urine samples collected during the clinic visit, total and high-density lipoprotein (HDL) cholesterol were assayed by the cholesterol oxidase method supplied by Boehringer Mannheim Diagnostics on a Roche COBAS Fara analyzer (Indianapolis, IN). Serum c-reactive protein was measured with a high-sensitivity immunoturbidimetric CRP-Latex assay (Kamiya Biomedical Company, Washington) and levels > 3 mg/L were defined as elevated. Urinary albumin was measured with the Dade Behring BN II nephelometer (Newark, Delaware). Serum and urine creatinine were measured using a multi-point enzymatic spectrophotometric assay on a Vitros 950 Ortho-Clinical Diagnostics analyzer (Raritan, New Jersey). Creatinine values were biochemically calibrated to Cleveland Clinic-equivalent Minnesota Beckman CX3 assay for analysis purposes [14]. eGFR was calculated via the Chronic Kidney Disease Epidemiology Collaboration (CKD-EPI) equation [1], and CKD was defined as ACR ≥ 30 mg/g or eGFR < 60 mL/min/1.73 m^2^.

During the clinic visit, BP was measured after a 5-min rest with a Hawksley random zero sphygmomanometer equipped with one of four cuff sizes selected following measurement of each participant’s arm circumference (Hawksley and Sons Ltd). The average of the 2 measures taken 1 min apart was used to define clinic BP. Upon completion of the study visit, participants were asked to complete an ABPM over the next 24 h. ABPM measurements were obtained with a portable, noninvasive oscillometric device (Spacelabs 90207; Medifacts International Ltd, Rockville, MD) with a cuff fitted to the participant’s non-dominant arm. Trained technicians instructed participants in the proper use of the ABPM device. With the participant in the seated position, 3–5 simultaneous ABPM and office sphygmomanometer BP readings were taken to calibrate the ABPM device. The device was programmed to measure BP every 20 min for 24 h, and participants were instructed to proceed with their normal daily activities but keep their arm still and extended at their side during each BP reading. Participants returned to the clinic after 24 h for the removal of the device. The monitor was connected to a computer and the BP readings were downloaded with commercially available software (Medicom, version 3.41; Medifacts Ltd). Quality control was assured by technician recertification, procedural checklists, and data review [12, 15–17].

### Assessment of 24-h BP variability

All BP readings were reviewed to eliminate out-of-range readings and errors due to motion artifacts or equipment problems, relying on predetermined acceptable ranges of SBP and diastolic BP (DBP) [17]. Twenty-four hour BP variability was defined by two metrics: day-night standard deviation (SD_dn_) and average real variability (ARV). SD_dn_ was calculated as a weighted average of the daytime and nighttime standard deviation of SBP and DBP, separately, during the ABPM period. The ARV was calculated for SBP and DBP, separately, as the mean of the absolute difference of consecutive BP measurements during the ABPM period.

### Statistical analysis

The current analysis was restricted to participants with valid ABPM data based on the International Database of Ambulatory Blood Pressure in relation to Cardiovascular Outcome (IDACO) criteria, which requires 10 daytime (defined as 10a-8p) and 5 nighttime (defined as 12p-6a) SBP and DBP measurements (*N* = 1,046). Participants missing clinic SBP (*n* = 5), with end-stage renal disease at baseline (*n* = 9), and without a serum creatinine measurement at baseline (*n* = 10) were excluded. We used multiple imputation (*n* = 10 data sets) and chained equations to impute ACR and other variables with missing data. Additional file 1: Table S1 summarizes the percentage of participants with missing data prior to imputation. We included 1,022 Jackson Heart Study participants in the current analysis.

Participant characteristics and SD_dn_ and ARV of SBP and DBP were calculated for those with and without CKD, separately. The statistical significance of differences across groups were calculated using t-tests and chi-square tests, as appropriate. Next, using linear regression, we calculated the adjusted differences in SD_dn_ and ARV of SBP and DBP for participants with, versus without, CKD. Three levels of adjustment were performed, using variables selected *a priori*. Initial models included adjustment for age and sex. A second model included age, sex, education, income, smoking status, waist circumference, diabetes, history of stroke, history of myocardial infarction, total cholesterol, HDL-cholesterol, c-reactive protein, statin use, and antihypertensive medication use. The full multivariable adjusted model included the variables in the second model and mean 24-h SBP in analyses of SD_dn_ and ARV of SBP, and mean 24-h DBP in analyses of SD_dn_ and ARV of DBP. Analyses were repeated comparing SD_dn_ and ARV of SBP and DBP between participants with eGFR less than versus greater than or equal to 60 ml/min/1.73 m^2^ and ACR greater than or equal to versus less than 30 mg/g. Finally, for individuals with CKD, we calculated differences in SD_dn_ and ARV of SBP and DBP associated with each study covariate included in full multivariable adjusted models. All analyses were conducted using Stata Version 13 (Stata Corp. College Station, TX).

## Results

### Participant characteristics

On average, compared to their counterparts without CKD, participants with CKD were older, had a larger waist circumference, a higher mean clinic and 24-h SBP and DBP, and were more likely to be taking an angiotensin-converting enzyme inhibitor or calcium channel blocker (Table 1). Also, participants with CKD had lower HDL-cholesterol compared to those without CKD. Those with CKD were more likely to have less than a high school education, diabetes, and a history of stroke.

**Table 1:** Characteristics of Jackson Heart Study participants with and without chronic kidney disease

	No CKD	CKD	*p*-value
	(*n* = 849)	(*n* = 173)	
Age, years	58.9 (0.4)	60.8 (0.9)	0.041
Female gender, %	68.3	65.1	0.452
Less than high school education, %	18.1	26.7	0.022
Low income, %	10.8	15.6	0.142
Diabetes, %	21.2	40.6	<0.001
History of stroke, %	2.9	8.8	0.002
History of myocardial infarction, %	4.1	7.5	0.074
Current smoking, %	5.3	7.4	0.329
Waist circumference, cm	99.1 (0.5)	104.5 (1.3)	<0.001
Total cholesterol, mg/dL	200.7 (1.4)	205.0 (3.5)	0.242
HDL-cholesterol, mg/dL	54.1 (0.5)	51.5 (1.0)	0.043
C-reactive protein > 3 mg/L, %	46.7	52.1	0.213
Mean clinic SBP, mmHg	126.0 (0.6)	132.9 (1.6)	<0.001
Mean clinic DBP, mmHg	77.3 (0.4)	77.1 (0.9)	0.781
Mean 24-h SBP, mmHg	125.0 (0.4)	132.7 (1.4)	<0.001
Mean 24-h DBP, mmHg	73.8 (0.3)	76.2 (0.9)	0.004
eGFR, mL/min/1.73 m^2^	94.4 (0.6)	77.7 (2.4)	<0.001
Albumin-to-creatinine ratio, mg/g	5.5 (3.7, 9.4)	55.3 (22.9, 125.5)	<0.001
Antihypertensive medication use, %			
Aldosterone antagonist	1.6	4.9	0.073
Alpha blocker	11.6	16.9	0.152
ACE inhibitor	35.8	47.0	0.035
Angiotensin II receptor blocker	12.2	13.3	0.753
Beta blocker	24.1	18.6	0.234
Calcium channel blocker	32.6	47.2	0.008
Diuretic	65.6	65.4	0.971
Renin inhibitor	NA	NA	---
Vasodilator	0.8	1.0	0.839

*CKD* chronic kidney disease, defined as an estimated glomerular filtration rate < 60 mL/min/1.73 m^2^ or an albumin-to-creatinine ratio ≥ 30 mg/g; *HDL* high-density lipoprotein, *SBP* systolic blood pressure, *DBP* diastolic blood pressure, *eGFR* estimated glomerular filtration rate, *ACE* angiotensin converting enzyme

Numbers in table are presented as mean (standard error) or percent except albumin-to-creatinine ratio, which is presented as median (interquartile range)

### CKD and SD_dn_ and ARV of SBP

SD_dn_ of SBP was higher among participants with versus without CKD (Table 2, top panel). After age and sex-adjustment and further adjustment for education, income, smoking status, waist circumference, diabetes, history of stroke, history of myocardial infarction, total cholesterol, HDL-cholesterol, c-reactive protein, statin use, and antihypertensive medication use, SD_dn_ of SBP was higher for participants with versus without CKD. The difference in SD_dn_ of SBP for those with, versus without, CKD was attenuated and not statistically significant after further adjustment for mean 24-h SBP. ARV of SBP was higher for those with versus without CKD after age and sex adjustment, but this difference was attenuated and no longer statistically significant after further multivariable adjustment.

**Table 2:** Association of chronic kidney disease status, albumin-to-creatinine ratio, and estimated glomerular filtration rate with measures of systolic blood pressure variability

*Chronic kidney disease status*			
Blood pressure variability measure	No CKD	CKD	*p*-value
	(*n* = 849)	(*n* = 173)	
Day-night standard deviation, mmHg			
Mean ± standard error	9.1 ± 0.1	10.2 ± 0.2	<0.001
Age, sex adjusted	0 (ref)	0.98 (0.59, 1.38)	<0.001
Multivariable adjusted 1^a^	0 (ref)	0.64 (0.19, 1.09)	0.005
Multivariable adjusted 2^b^	0 (ref)	0.31 (−0.12, 0.74)	0.162
Average real variability, mmHg			
Mean ± standard error	8.6 ± 0.1	9.2 ± 0.2	0.001
Age, sex adjusted	0 (ref)	0.52 (0.18, 0.86)	0.003
Multivariable adjusted 1^a^	0 (ref)	0.32 (−0.09, 0.72)	0.125
Multivariable adjusted 2^b^	0 (ref)	0.08 (−0.31, 0.46)	0.695
*Albumin-to-creatinine ratio, mg/g*			
Blood pressure variability measure	ACR < 30	ACR ≥ 30	*p*-value
	(*n* = 885)	(*n* = 137)	
Day-night standard deviation, mmHg			
Mean ± standard error	9.2 ± 0.1	10.2 ± 0.3	<0.001
Age, sex adjusted	0 (ref)	1.09 (0.65, 1.53)	<0.001
Multivariable adjusted 1^a^	0 (ref)	0.85 (0.33, 1.37)	0.001
Multivariable adjusted 2^b^	0 (ref)	0.35 (−0.16, 0.85)	0.179
Average real variability, mmHg			
Mean ± standard error	8.7 ± 0.1	9.2 ± 0.2	0.01
Age, sex adjusted	0 (ref)	0.57 (0.18, 0.95)	0.004
Multivariable adjusted 1^a^	0 (ref)	0.41 (−0.06, 0.89)	0.085
Multivariable adjusted 2^b^	0 (ref)	0.05 (−0.41, 0.50)	0.844
*Estimated glomerular filtration rate, mL/min/1.73 m* ^*2*^			
Blood pressure variability measure	eGFR ≥ 60	eGFR < 60	*p*-value
	(*n* = 960)	(*n* = 62)	
Day-night standard deviation, mmHg			
Mean ± standard error	9.3 ± 0.1	10.4 ± 0.4	<0.001
Age, sex adjusted	0 (ref)	0.58 (−0.04, 1.20)	0.066
Multivariable adjusted 1^a^	0 (ref)	0.24 (−0.39, 0.86)	0.455
Multivariable adjusted 2^b^	0 (ref)	0.17 (−0.42, 0.77)	0.565
Average real variability, mmHg			
Mean ± standard error	8.7 ± 0.1	9.5 ± 0.2	0.004
Age, sex adjusted	0 (ref)	0.27 (−0.24, 0.78)	0.296
Multivariable adjusted 1^a^	0 (ref)	−0.01 (−0.54, 0.52)	0.981
Multivariable adjusted 2^b^	0 (ref)	−0.05 (−0.56, 0.46)	0.842

*CKD* chronic kidney disease, *ACR* albumin-to-creatinine ratio, *eGFR* estimated glomerular filtration rate

^a^Adjusted for age, sex, education, income, smoking status, waist circumference, diabetes, history of stroke, history of myocardial infarction, total cholesterol, high-density lipoprotein cholesterol, c-reactive protein, statin use, and antihypertensive medication use

^b^Adjusted for above model 1 plus mean 24-h systolic blood pressure

SD_dn_ and ARV of SBP were higher among participants with ACR ≥ 30 mg/g versus their counterparts with ACR < 30 mg/g (Table 2, middle panel). These associations were attenuated and no longer statistically significant after multivariable adjustment including mean 24-h SBP. SD_dn_ and ARV of SBP were higher for participants with eGFR < 60 mL/min/1.73 m^2^ compared to their counterparts with eGFR ≥ 60 mL/min/1.73 m^2^ (Table 2, bottom panel). These differences were attenuated and no longer statistically significant after adjustment for age and sex or further multivariable adjustment.

Among participants with CKD, older age, total cholesterol, and higher 24-h SBP were associated with higher SD_dn_ and ARV of SBP (Additional file 2: Table S2). Female gender and alpha blocker use were associated with higher SD_dn_ of SBP but not ARV of SBP. Higher HDL-cholesterol was associated with lower ARV of SBP among those with CKD.

### CKD and SD_dn_ and ARV of DBP

SD_dn_ and ARV of DBP were higher for participants with versus without CKD (Table 3, top panel). SD_dn_ of DBP was higher for participants with versus without CKD after age, sex-adjustment and after further adjustment for education, income, smoking status, waist circumference, diabetes, history of stroke, history of myocardial infarction, total and HDL-cholesterol, c-reactive protein, statin use, and antihypertensive medication use. However, this association was attenuated and not statistically significant after adjustment for mean 24-h DBP. The age and sex-adjusted mean difference in ARV of DBP was higher for individuals with CKD compared to those without CKD, but this association was no longer statistically significant after further multivariable adjustment. Compared to those with ACR < 30 mg/g, participants with ACR ≥ 30 mg/g had higher SD_dn_ of DBP and ARV of DBP (Table 3, middle panel). This association was no longer statistically significant after multivariable adjustment including mean 24-h DBP. Differences in SD_dn_ of DBP and ARV of DBP between participants with eGFR < and ≥ 60 mL/min/1.73 m^2^ were not statistically significant before or after multivariable adjustment (Table 3, bottom panel).

**Table 3:** Association of chronic kidney disease status, albumin-to-creatinine ratio, and estimated glomerular filtration rate with measures of diastolic blood pressure variability

*Chronic kidney disease status*			
Blood pressure variability measure	No CKD	CKD	*p*-value
	(*n* = 849)	(*n* = 173)	
Day-night standard deviation, mmHg			
Mean ± standard error	8.0 ± 0.1	8.5 ± 0.2	0.004
Age, sex adjusted	0 (ref)	0.54 (0.17, 0.90)	0.004
Multivariable adjusted 1^a^	0 (ref)	0.43 (0.00, 0.85)	0.049
Multivariable adjusted 2^b^	0 (ref)	0.25 (−0.16, 0.66)	0.231
Average real variability, mmHg			
Mean ± standard error	7.5 ± 0.1	7.9 ± 0.2	0.043
Age, sex adjusted	0 (ref)	0.37 (0.01, 0.72)	0.041
Multivariable adjusted 1^a^	0 (ref)	0.32 (−0.09, 0.72)	0.128
Multivariable adjusted 2^b^	0 (ref)	0.19 (−0.21, 0.59)	0.351
*Albumin-to-creatinine ratio, mg/g*			
Blood pressure variability measure	ACR < 30	ACR ≥ 30	*p*-value
	(*n* = 885)	(*n* = 137)	
Day-night standard deviation, mmHg			
Mean ± standard error	8.0 ± 0.1	8.6 ± 0.2	0.002
Age, sex adjusted	0 (ref)	0.64 (0.23, 1.06)	0.002
Multivariable adjusted 1^a^	0 (ref)	0.63 (0.13, 1.13)	0.013
Multivariable adjusted 2^b^	0 (ref)	0.39 (−0.10, 0.87)	0.122
Average real variability, mmHg			
Mean ± standard error	7.5 ± 0.1	8.1 ± 0.2	0.011
Age, sex adjusted	0 (ref)	0.53 (0.13, 0.93)	0.010
Multivariable adjusted 1^a^	0 (ref)	0.57 (0.09, 1.05)	0.021
Multivariable adjusted 2^b^	0 (ref)	0.39 (−0.09, 0.87)	0.108
*Estimated glomerular filtration rate, mL/min/1.73 m* ^*2*^			
Blood pressure variability measure	eGFR ≥ 60	eGFR < 60	*p*-value
	(*n* = 960)	(*n* = 62)	
Day-night standard deviation, mmHg			
Mean ± standard error	8.1 ± 0.1	8.2 ± 0.3	0.572
Age, sex adjusted	0 (ref)	0.17 (−0.39, 0.72)	0.551
Multivariable adjusted 1^a^	0 (ref)	0.07 (−0.50, 0.64)	0.805
Multivariable adjusted 2^b^	0 (ref)	0.01 (−0.55, 0.56)	0.977
Average real variability, mmHg			
Mean ± standard error	7.6 ± 0.1	7.5 ± 0.2	0.655
Age, sex adjusted	0 (ref)	−0.13 (−0.66, 0.41)	0.641
Multivariable adjusted 1^a^	0 (ref)	−0.19 (−0.75, 0.36)	0.487
Multivariable adjusted 2^b^	0 (ref)	−0.24 (−0.78, 0.30)	0.384

*CKD* chronic kidney disease, *ACR* albumin-to-creatinine ratio, *eGFR* estimated glomerular filtration rate

^a^Adjusted for age, sex, education, income, smoking status, waist circumference, diabetes, history of stroke, history of myocardial infarction, total cholesterol, high-density lipoprotein cholesterol, c-reactive protein, statin use, and antihypertensive medication use

^b^Adjusted for above model 1 plus mean 24-h diastolic blood pressure

Among participants with CKD, older age, larger waist circumference and higher mean 24-h DBP were associated with higher SD_dn_ and ARV of DBP (Additional file 3: Table S3). Female gender was associated with lower ARV of DBP but not SD_dn_ of DBP among those with CKD.

## Discussion

This population-based study of African American adults suggests an association between CKD and higher SD_dn_ of BP and ARV of DBP. However, these associations were explained by the higher mean 24-h BP among participants with CKD. Older age, larger waist circumference, and higher mean 24-h SBP were associated with higher 24-h SBP variability. Female gender, larger waist circumference, and higher mean 24-h DBP were associated with higher 24-h DBP variability.

Twenty-four hour BP variability has been associated with adverse outcomes and may represent a novel CVD risk factor. In a pooled analysis of 11 studies, Hansen, et al. reported that ARV of BP was associated with total and cardiovascular mortality (multivariable adjusted hazard ratio [HR] for ARV of SBP: 1.11 (95 % confidence interval [CI]: 1.04–1.18) and 1.17 (95 % CI: 1.07–1.28), respectively; multivariable adjusted HR for ARV of DBP: 1.13 (95 % CI: 1.07–1.19) and 1.21 (95 % CI: 1.12–1.31), respectively) [4]. Additionally, Eguchi, et al. reported SD of nighttime SBP to be an independent risk factor for the composite outcome of stroke, myocardial infarction, or sudden cardiac death (HR: 2.21; 95 % CI 1.08–4.53) [18].

Long-term BP variability (i.e. “visit-to-visit” variability) has been associated with adverse outcomes among individuals with CKD in several studies [19–22]. For example, in an analysis of 374 elderly patients with CKD, DiIorio, et al. reported that each 1 % increase in visit-to-visit BP variability was associated with a higher risk for all-cause mortality (HR: 1.05 (95 % CI: 1.02–1.09) [19]. In an analysis of the African American Study of Kidney Disease (AASK) trial, McMullan, et al. found an association between visit-to-visit BP variability with all-cause mortality and cardiovascular mortality (HR comparing highest with lowest tertile of visit-to-visit BP variability: 2.82 (95 % CI: 1.14–6.95) and 4.91 (95 % CI: 1.12–21.50), respectively) [20]. Although not extensively studied, 24-h BP variability has been associated with target organ damage among individuals with CKD in at least one study. Ryu, et al. reported that ARV of SBP was associated with left ventricular hypertrophy (odds ratio: 1.05 (95 % CI: 1.02–1.09)), but not kidney injury (defined as eGFR <30 mL/min/1.73 m^2^ and proteinuria) in a large sample of hypertensive CKD patients in Korea [23].

In the current analysis, CKD was associated with higher 24-h BP variability, but this association was no longer present after adjustment for mean 24-h BP. The direction of the association between mean BP and BP variability is unclear. For example, higher mean BP and its associated sequelae (e.g., arterial stiffness) could lead to higher BP variability. Alternatively, higher BP variability could cause vascular injury, making BP harder to control. Given the cross-sectional nature of our analysis, future studies with longitudinal ABPM assessments could prove helpful for characterizing this association. Autonomic imbalance is a characteristic of BP variability which has also been associated with CKD progression [24] and may suggest a potential physiologic mechanism for the association between 24-h variability and CKD. Sympathetic nerve terminals innervate the kidneys directly, potentially affecting tubular function by enhancing solute and fluid resorption and modifying renal microvascular function by enhancing the effects of angiotensin [24–26]. Additionally, several CVD risk factors which adversely affect the glomerulus (e.g. endothelial dysfunction, dyslipidemia, insulin resistance, and oxidative stress) are also associated with high sympathetic and low parasympathetic tone, suggesting a potential mechanism involving the nervous system’s role in regulating hemodynamics, vascular tone, metabolism, and inflammation [24].

### Strengths and limitations

Our study maintains several strengths, including the large sample size of African American adults with available ABPM data and the availability of comprehensive data which allowed us to adjust for potential confounders. However, the current findings should be considered in the context of certain limitations. The current analysis employed a cross-sectional study design. Also, eGFR and albuminuria were only assessed at a single time point and therefore, misclassification of CKD status may be present. We were not able to assess the effect of plasma metanephrines, a marker of adrenergic activation, on 24-h BP variability. Finally, the applicability of our results to race/ethnic groups beyond African Americans needs to be assessed in other studies.

## Conclusions

In conclusion, data from the current study suggest that CKD may be associated with higher SD_dn_ of SBP and DBP and ARV of DBP over 24-h. However, these associations were no longer present after adjustment for 24-h mean BP. Further studies with repeated measurements of 24-h BP variability and mean BP are needed to determine the direction of the association. Such data may be useful in identifying new therapeutic targets in an effort to improve health outcomes for individuals with CKD.

## Additional files

Additional file 1: Table S1. Summary of missing data prior to multiple imputation. Additional file 2: Table S2. Factors associated with systolic blood pressure variability among participants with chronic kidney disease. Additional file 3: Table S3. Factors associated with diastolic blood pressure variability among participants with chronic kidney disease.

## Abbreviations

ABPM: Ambulatory blood pressure monitoring
ACR: Albumin-to-creatinine ratio
ARV: Average real variability
BP: Blood pressure
CI: Confidence interval
CKD: Chronic kidney disease
CVD: Cardiovascular disease
DBP: Diastolic blood pressure
eGFR: Estimated glomerular filtration rate
HDL: High-density lipoprotein
HR: Hazard ratio
SBP: Systolic blood pressure
SD_dn_: Day-night standard deviation
