# Supplementary material for: Association between 24-hour blood pressure variability and chronic kidney disease: a cross-sectional analysis of African Americans participating in the Jackson heart study
Source: BMC Nephrol. 2015 Jun 18;16:84. doi: 10.1186/s12882-015-0085-6 (PMC4477603; doi:10.1186/s12882-015-0085-6)
Supplement: Additional file 1: Table S1. — Summary of missing data prior to multiple imputation. [file 12882_2015_85_MOESM1_ESM.docx]

Additional file 1: Table S1. Summary of missing data prior to multiple imputation.

| Characteristics | N (%) missing |
| --- | --- |
| Age, years | 0 (0%) |
| Female gender, % | 0 (0%) |
| Less than high school education, % | 4 (0.4%) |
| Low income, % | 117 (11.4%) |
| Diabetes, % | 1 (0.1%) |
| History of stroke, % | 0 (0%) |
| History of myocardial infarction, % | 0 (0%) |
| Current smoking, % | 0 (0%) |
| Waist circumference, cm | 2 (0.2%) |
| Total cholesterol, mg/dL | 67 (6.6%) |
| HDL-cholesterol, mg/dL | 68 (6.7%) |
| C-reactive protein > 3 mg/L, % | 2 (0.2%) |
| Mean clinic SBP, mmHg | 0 (0%) |
| Mean clinic DBP, mmHg | 0 (0%) |
| Mean 24-hour SBP, mmHg | 0 (0%) |
| Mean 24-hour DBP, mmHg | 0 (0%) |
| eGFR, mL/min/1.73m^2^ | 0 (0%) |
| Albumin-to-creatinine ratio, mg/g | 243 (23.8%) |

HDL: high-density lipoprotein; SBP: systolic blood pressure; DBP: diastolic blood pressure; eGFR: estimated glomerular filtration rate
