# Supplementary material for: Association between 24-hour blood pressure variability and chronic kidney disease: a cross-sectional analysis of African Americans participating in the Jackson heart study
Source: BMC Nephrol. 2015 Jun 18;16:84. doi: 10.1186/s12882-015-0085-6 (PMC4477603; doi:10.1186/s12882-015-0085-6)
Supplement: Additional file 2: Table S2. — Factors associated with systolic blood pressure variability among participants with chronic kidney disease. [file 12882_2015_85_MOESM2_ESM.docx]

Additional file 2: Table S2. Factors associated with systolic blood pressure variability among participants with chronic kidney disease

| Characteristic | Day-night standard deviation  β-coefficient (95% CI) | p-value | Average real variability  β-coefficient (95% CI) | p-value |
| --- | --- | --- | --- | --- |
| Age, per 10 years | 0.41 (0.20, 0.62) | <0.001 | 0.47 (0.29, 0.65) | <0.001 |
| Female gender | -0.54 (-1.00, -0.07) | 0.024 | -0.34 (-0.74, 0.05) | 0.089 |
| Less than high school education | 0.03 (-0.44, 0.51) | 0.889 | -0.11 (-0.52, 0.29) | 0.585 |
| Low income | 0.08 (-0.52, 0.68) | 0.797 | 0.27 (-0.24, 0.78) | 0.295 |
| Current smoking | 0.16 (-0.72, 1.03) | 0.720 | 0.46 (-0.28, 1.20) | 0.222 |
| Waist circumference, per 15 cm | 0.11 (-0.11, 0.32) | 0.310 | 0.17 (-0.01, 0.35) | 0.059 |
| Diabetes | -0.17 (-0.61, 0.27) | 0.456 | 0.20 (-0.18, 0.57) | 0.298 |
| History of stroke | 0.07 (-0.76, 0.90) | 0.867 | -0.04 (-0.75, 0.66) | 0.908 |
| History of myocardial infarction | 0.52 (-0.35, 1.38) | 0.240 | 0.23 (-0.50, 0.96) | 0.539 |
| Total cholesterol, per 40 mg/dL | 0.25 (0.05, 0.45) | 0.014 | 0.18 (0.01, 0.35) | 0.043 |
| HDL-cholesterol, per 15 mg/dL | -0.18 (-0.39, 0.04) | 0.106 | -0.21 (-0.40, -0.03) | 0.021 |
| C-reactive protein > 3 mg/L | -0.12 (-0.52, 0.29) | 0.571 | -0.08 (-0.42, 0.27) | 0.663 |
| Statin use | 0.44 (-0.04, 0.93) | 0.074 | 0.08 (-0.33, 0.49) | 0.705 |
| Antihypertensive medication use | 0.09 (-0.70, 0.87) | 0.822 | 0.17 (-0.53, 0.87) | 0.638 |
| Aldosterone antagonist use | -0.69 (-1.97, 0.59) | 0.293 | -0.21 (-1.30, 0.88) | 0.704 |
| Alpha blocker use | 0.82 (0.24, 1.40) | 0.006 | 0.35 (-0.14, 0.84) | 0.165 |
| ACE inhibitor use | 0.41 (-0.02, 0.84) | 0.064 | 0.25 (-0.12, 0.61) | 0.181 |
| Angiotensin II receptor blocker use | 0.52 (-0.08, 1.13) | 0.089 | 0.38 (-0.13, 0.90) | 0.144 |
| Beta blocker use | -0.10 (-0.57, 0.37) | 0.676 | 0.06 (-0.34, 0.45) | 0.783 |
| Calcium channel blocker use | 0.02 (-0.39, 0.44) | 0.920 | -0.27 (-0.62, 0.08) | 0.131 |
| Diuretic use | -0.12 (-0.53, 0.29) | 0.568 | -0.23 (-0.58, 0.12) | 0.192 |
| Vasodilator use | -0.95 (-3.00, 1.11) | 0.365 | 0.14 (-1.60, 1.88) | 0.878 |
| 24-hour systolic blood pressure, per 15 mmHg | 0.84 (0.61, 1.07) | <0.001 | 0.65 (0.46, 0.84) | <0.001 |

CI: confidence interval

For each column above, all variables were included in a single multivariable model. Units for continuous variables represent one standard deviation.
