# Supplementary material for: Association between 24-hour blood pressure variability and chronic kidney disease: a cross-sectional analysis of African Americans participating in the Jackson heart study
Source: BMC Nephrol. 2015 Jun 18;16:84. doi: 10.1186/s12882-015-0085-6 (PMC4477603; doi:10.1186/s12882-015-0085-6)
Supplement: Additional file 3: Table S3. — Factors associated with diastolic blood pressure variability among participants with chronic kidney disease. [file 12882_2015_85_MOESM3_ESM.docx]

Additional file 3: Table S3. Factors associated with diastolic blood pressure variability among participants with chronic kidney disease

| Characteristic | Day-night standard deviation  β-coefficient (95% CI) | p-value | Average real variability  β-coefficient (95% CI) | p-value |
| --- | --- | --- | --- | --- |
| Age, per 10 years | 0.24 (0.04, 0.44) | 0.017 | 0.24 (0.04, 0.43) | 0.019 |
| Female gender | -0.45 (-0.90, 0.00) | 0.050 | -0.53 (-0.97, -0.08) | 0.020 |
| Less than high school education | -0.17 (-0.61, 0.27) | 0.443 | -0.23 (-0.67, 0.20) | 0.286 |
| Low income | 0.39 (-0.18, 0.96) | 0.182 | 0.26 (-0.29, 0.81) | 0.355 |
| Current smoking | 0.24 (-0.56, 1.05) | 0.553 | 0.36 (-0.43, 1.15) | 0.373 |
| Waist circumference, per 15 cm | 0.57 (0.37, 0.76) | <0.001 | 0.55 (0.36, 0.74) | <0.001 |
| Diabetes | -0.22 (-0.61, 0.18) | 0.276 | 0.05 (-0.33, 0.44) | 0.790 |
| History of stroke | -0.25 (-1.01, 0.52) | 0.522 | -0.37 (-1.13, 0.38) | 0.329 |
| History of myocardial infarction | 0.10 (-0.69, 0.89) | 0.809 | -0.28 (-1.06, 0.50) | 0.476 |
| Total cholesterol, per 40 mg/dL | 0.07 (-0.12, 0.26) | 0.449 | 0.01 (-0.18, 0.19) | 0.938 |
| HDL-cholesterol, per 15 mg/dL | -0.06 (-0.26, 0.13) | 0.511 | -0.15 (-0.34, 0.38) | 0.110 |
| C-reactive protein > 3 mg/L | -0.09 (-0.46, 0.28) | 0.648 | 0.02 (-0.34, 0.38) | 0.909 |
| Statin use | -0.03 (-0.48, 0.42) | 0.895 | -0.06 (-0.50, 0.38) | 0.796 |
| Antihypertensive medication use | -0.37 (-1.11, 0.38) | 0.335 | -0.31 (-1.05, 0.43) | 0.414 |
| Aldosterone antagonist use | -1.13 (-2.30, 0.05) | 0.061 | -1.15 (-2.31, 0.01) | 0.053 |
| Alpha blocker use | 0.39 (-0.14, 0.92) | 0.147 | -0.30 (-0.82, 0.22) | 0.261 |
| ACE inhibitor use | 0.32 (-0.07, 0.72) | 0.110 | 0.08 (-0.31, 0.47) | 0.681 |
| Angiotensin II receptor blocker use | 0.21 (-0.34, 0.77) | 0.454 | 0.15 (-0.39, 0.70) | 0.582 |
| Beta blocker use | -0.22 (-0.65, 0.21) | 0.320 | -0.25 (-0.67, 0.17) | 0.254 |
| Calcium channel blocker use | -0.30 (-0.67, 0.08) | 0.121 | -0.25 (-0.62, 0.12) | 0.186 |
| Diuretic use | -0.17 (-0.55, 0.21) | 0.377 | -0.25 (-0.62, 0.12) | 0.190 |
| Vasodilator use | -0.16 (-2.05, 1.72) | 0.863 | -0.25 (-0.62, 0.12) | 0.794 |
| 24-hour diastolic blood pressure, per 10 mmHg | 0.64 (0.42, 0.86) | <0.001 | 0.54 (0.32, 0.76) | <0.001 |

CI: confidence interval

For each column above, all variables were included in a single multivariable model. Units for continuous variables represent one standard deviation.
